# Accuracy of circulating histones in predicting persistent organ failure and mortality in patients with acute pancreatitis

**DOI:** 10.1002/bjs.10538

**Published:** 2017-04-24

**Authors:** T Liu, W Huang, P Szatmary, S T Abrams, Y Alhamdi, Z Lin, W Greenhalf, G Wang, R Sutton, C H Toh

**Affiliations:** Institute of Infection and Global Health, University of Liverpool, Liverpool, UK; National Institute for Health Research (NIHR) Liverpool Pancreas Biomedical Research Unit, Royal Liverpool University Hospital, Liverpool, UK; Roald Dahl Haemostasis and Thrombosis Centre, Royal Liverpool University Hospital, Liverpool, UK; Department of Integrated Traditional Chinese and Western Medicine, Sichuan Provincial Pancreatitis Centre, West China Hospital, Sichuan University, Chengdu, China

## Abstract

**Background:**

Early prediction of acute pancreatitis severity remains a challenge. Circulating levels of histones are raised early in mouse models and correlate with disease severity. It was hypothesized that circulating histones predict persistent organ failure in patients with acute pancreatitis.

**Methods:**

Consecutive patients with acute pancreatitis fulfilling inclusion criteria admitted to Royal Liverpool University Hospital were enrolled prospectively between June 2010 and March 2014. Blood samples were obtained within 48 h of abdominal pain onset and relevant clinical data during the hospital stay were collected. Healthy volunteers were enrolled as controls. The primary endpoint was occurrence of persistent organ failure. The predictive values of circulating histones, clinical scores and other biomarkers were determined.

**Results:**

Among 236 patients with acute pancreatitis, there were 156 (66·1 per cent), 57 (24·2 per cent) and 23 (9·7 per cent) with mild, moderate and severe disease respectively, according to the revised Atlanta classification. Forty-seven healthy volunteers were included. The area under the receiver operating characteristic (ROC) curve (AUC) for circulating histones in predicting persistent organ failure and mortality was 0·92 (95 per cent c.i. 0·85 to 0·99) and 0·96 (0·92 to 1·00) respectively; histones were at least as accurate as clinical scores or biochemical markers. For infected pancreatic necrosis and/or sepsis, the AUC was 0·78 (0·62 to 0·94). Histones did not predict or correlate with local pancreatic complications, but correlated negatively with leucocyte cell viability (*r* = –0·511, *P* = 0·001).

**Conclusion:**

Quantitative assessment of circulating histones in plasma within 48 h of abdominal pain onset can predict persistent organ failure and mortality in patients with acute pancreatitis. Early death of immune cells may contribute to raised circulating histone levels in acute pancreatitis.

## Teaser

Potential as early biomarker

## Introduction

Acute pancreatitis is one of the leading gastrointestinal disorders that require urgent clinical care and is increasing in incidence^1^. The clinical course of acute pancreatitis is variable, ranging from mild (uneventful clinical course), through moderate (local complication or transient organ failure) to severe (occurrence of persistent organ failure) disease^2,3^. Infected pancreatic necrosis^4^ and/or sepsis^5^ are major complications contributing to death at any stage. However, the principal cause of early death is persistent organ failure^5^. Early recognition of patients at risk of persistent organ failure is critical to guide fluid resuscitation and initiate high-dependency or intensive care treatment, and reduce morbidity and mortality^6^. Indeed, early stratification of disease severity improves clinical outcomes and significantly reduces length of hospital stay^7^.

Improvements in imaging, such as CT, have not proven superior to clinical scoring systems in early prediction of acute pancreatitis severity^8^. A recent multicentre study^9^ has shown that existing clinical scores such as the Systemic Inflammatory Response Syndrome (SIRS) score, Bedside Index for Severity in Acute Pancreatitis (BISAP), Acute Physiology And Chronic Health Examination (APACHE) II and Sequential Organ Failure Assessment (SOFA), either alone or in combination, are of limited clinical use for predicting persistent organ failure. The best predictor had a sensitivity and specificity of only 75 per cent on or within 48 h of admission. The latest meta-analyses concluded that there is no adequate predictor of persistent organ failure within 48 h of hospital admission^10^, or justifiable prediction models for mortality^11^. There is thus a pressing need for the identification and development of more powerful predictive markers.

**Fig. 1:**
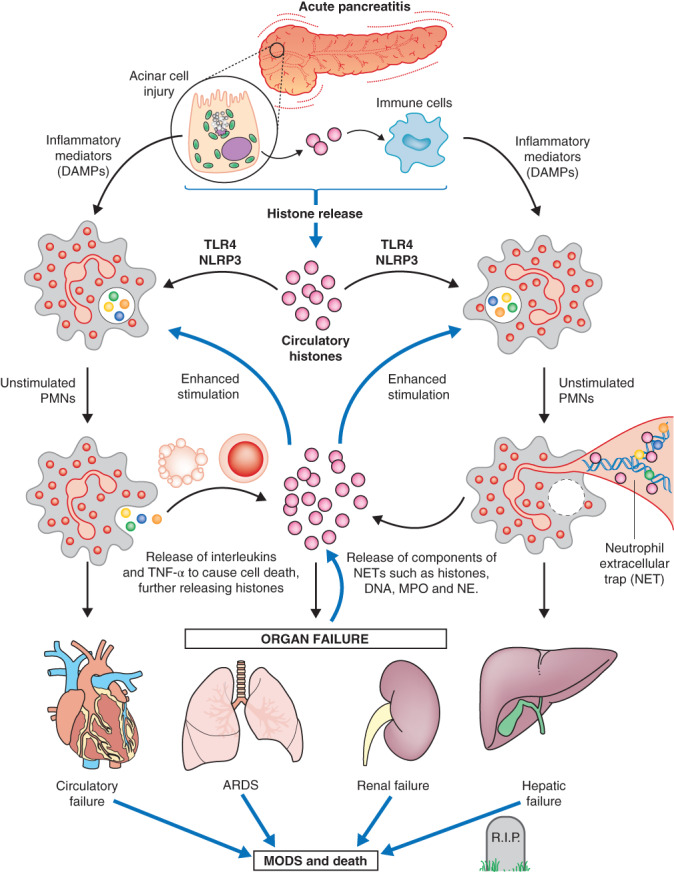
Proposed role of extracellular histones in acute pancreatitis. After injury caused by pancreatic toxins, the pancreas (primarily pancreatic acinar cells) releases damage-associated molecular pattern molecules including extracellular histones. The released extracellular histones and other inflammatory mediators stimulate resident immune cells to release more proinflammatory cytokines/chemokines. Once released into the circulation, the circulating histones activate polymorphonuclear neutrophils (PMNs) and facilitate neutrophil extracellular trap (NET) formation. Apoptotic and necrotic neutrophils and NETs release more histones and inflammatory mediators, which further stimulate PMN infiltration and NET formation. Stimulated PMNs, NET components, circulating histones and other inflammatory mediators cause distant organ dysfunction, such as acute respiratory distress syndrome or even multiple organ dysfunction syndrome (MODS). MODS in turn causes excessive release of circulating histones and other inflammatory mediators, triggering a vicious cycle of uncontrolled MODS, coagulation and death. TLR, Toll-like receptor; NLRP, NOD-like receptor family, pyrin domain-containing; IL, interleukin; TNF, tumour necrosis factor; MPO, myeloperoxidase; NE, neutrophil elastase

Recently, damage-associated molecular pattern molecules (DAMPs)^12^, such as high-mobility group box 1 (HMGB1)^13^, cell-free DNA^14^, nucleosomes^15^ and histones^16^, have been investigated in experimental acute pancreatitis models, and most have shown a correlation between circulating levels and acute pancreatitis severity^12,17^. Furthermore, levels of HMGB1^18^, nucleosomes^17^ and cell-free DNA^19^ have been associated with organ failure in human acute pancreatitis. The proposed role of these DAMPs in acute pancreatitis is illustrated in *Fig. 1*. Histones are well conserved nuclear proteins that are essential for DNA packaging and gene regulation. During tissue damage and cell death, nuclear chromatin is cleaved and released outside the cell where it is degraded into individual histones^20^. Circulating histones, the most abundant nuclear proteins, are barely detectable in the blood unless there is extensive cell death, such as in severe sepsis^21,22^ and trauma^23^. A recent review^12^ has suggested that circulating histones act as DAMPs that cause sterile inflammation and contribute to SIRS and organ failure. Extracellular histones are also toxic to endothelial cells^21,23^, platelets^24^ and leucocytes^25^. Furthermore, they have been reported to activate coagulation and stimulate cytokine release^23,26^. In mouse models, histone infusion causes death by multiple organ failure, which can be rescued by antihistone antibodies^21,23,27^. Clinically, high levels of circulating histones have been found in patients with severe blunt trauma^23^ and sepsis^22^, and have been associated with the development of respiratory failure^23^, new-onset cardiac complications^22^ and thrombocytopenia^28^.

The authors^16^ have demonstrated previously that circulating histone levels rise very early in mouse acute pancreatitis models, and are strongly associated with disease severity and distant organ injury. Therefore, the hypothesis for the present study was that plasma levels of circulating histones may have early predictive value for persistent organ failure and other major clinical outcomes in patients with acute pancreatitis.

## Methods

This study was designed, conducted and reported according to STROBE guidance^29^ for observational studies. A consecutive cohort of patients with acute pancreatitis admitted to the Royal Liverpool University Hospital between June 2010 and March 2014 were enrolled once written informed consent had been obtained. Inclusion criteria were: first episode of acute pancreatitis as defined by the revised Atlanta classification^2^; and availability of blood samples within 24 h of admission. Exclusion criteria were: age below 18 or over 85 years; advanced pulmonary, cardiac, renal diseases (chronic kidney disease stage 4–5), liver cirrhosis (modified Child–Pugh grade 2–3) or malignancy; pregnancy, chronic pancreatitis or trauma as the aetiology; and duration of abdominal pain before admission exceeding 24 h or referral from other hospitals. A group of healthy volunteers was also included.

The study protocols and acute pancreatitis biobank were approved by local research ethics committees (reference: 10/H1308/31).

### Sample and data collection

Peripheral blood samples were collected within 24 h of admission (within 48 h of onset of abdominal pain). Serum (serum separator tube) and plasma (EDTA tube) were obtained after centrifugation at 1500 *g* for 10 min. Leucocytes were freshly isolated from whole blood and cell viability assessed using 0·1 per cent trypan blue (Life Technologies, Warrington, UK) and a Countess™ automated cell counter (Invitrogen, Glasgow, UK). Samples were stored at –80°C before use. Collection, processing, storage, monitoring and use of samples followed standard operating procedures (SOPs) and good clinical laboratory practice. Demographic and clinical data were recorded prospectively and maintained in an electronic database in accordance with SOPs. SIRS, BISAP, APACHE II and SOFA scores were calculated within 24 h of admission^9^. The first and the worst modified CT severity index (MCTSI)^8^ values were enumerated using contrast-enhanced CT images.

### Outcomes of interest

The primary outcome of persistent organ failure was defined by a modified SOFA score of at least 2 for 48 h or more that manifested in failure of at least one of the respiratory, cardiovascular or renal systems^3^. In patients with pre-existing chronic kidney disease (stage 1–3), a 2-point worsening of kidney function, based on the estimated glomerular filtration rate^30^, was used to diagnose renal failure regardless of serum creatinine levels. Local complications were defined according to the revised Atlanta classfication^2^. Major infection was defined as the appearance of either infected pancreatic necrosis, sepsis or both, at least 3 days after admission. Mortality was recorded for the index hospital admission.

### Clinical biomarker analysis

Plasma histone levels were determined by quantitative western blotting^22,23,27,28^, with intra-assay and inter-assay variability of 4·6 and 4·3 per cent respectively. Plasma interleukin (IL)-6 and IL-8 (R&D, Abingdon, UK) were measured by enzyme-linked immunosorbent assay, in accordance with the manufacturer's instructions. Haematocrit, urea, creatinine, C-reactive protein (CRP) and other routine clinical biomarkers were reported by the Department of Clinical Biochemistry of the hospital.

### Statistical analysis

Continuous data are reported as median (i.q.r.). Continuous variables were compared by Mann–Whitney *U* test (2 groups) and Kruskal–Wallis test (3 or more groups). Categorical data were compared by means of χ^2^ or Fisher's exact tests. Spearman rank correlation was used for correlation analysis.

Receiver operating characteristic (ROC) curves were constructed for predictive variables, and the area under the curve (AUC) with 95 per cent confidence intervals (c.i.) calculated. Optimal cut-off values for sensitivity, specificity, positive predictive value (PPV), negative predictive value (NPV), positive likelihood ratio (PLR) and negative likelihood ratio (NLR) for each parameter were derived from the ROC curves. Post-test probability was obtained from the prevalence outcomes of interest and PLR. All tests were two-tailed and statistical significance was set at *P* < 0·050. The analyses were performed using SPSS® version 22.0 (IBM, Armonk, New York, USA).

## Results

**Fig. 2:**
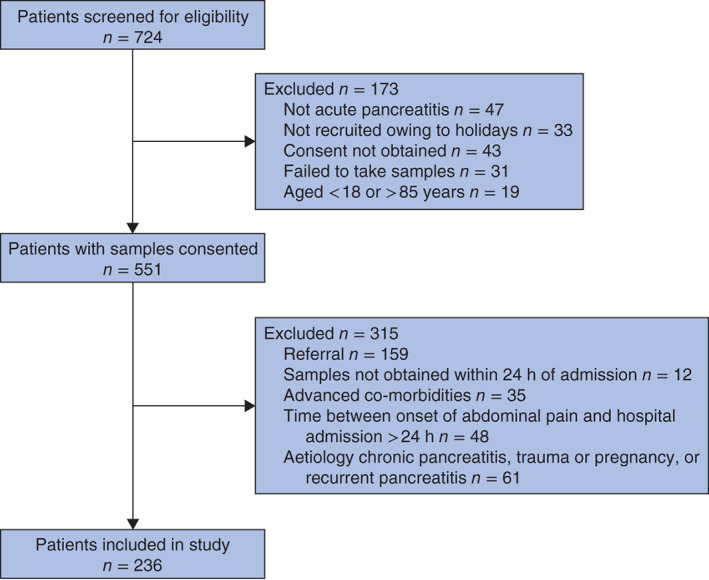
Flow chart showing patient selection

A total of 236 consecutive patients with pancreatitis (mild: 156, 66·1 per cent; moderate: 57, 24·2 per cent; severe: 23, 9·7 per cent) fulfilling the inclusion criteria were included in this study (*Fig. 2*). Baseline characteristics and clinical outcomes for each group are outlined in *Table 1*. Twenty-three patients (9·7 per cent) developed persistent organ failure; this occurred within 24 h of admission in 11 of these patients (*Fig. S1*, supporting information). Fifteen patients (6·4 per cent) had transient organ failure without local complications. Sixty patients (25·4 per cent) developed local complications; acute peripancreatic and acute necrotic collection each had an incidence of 12·7 per cent (30 patients). Major infection occurred in nine patients (3·8 per cent); there was one instance of infected pancreatic necrosis in the moderate group and eight in the severe group. Nine patients died (3·8 per cent), all of whom had severe pancreatitis.

**Table 1:** Demographic and clinical outcomes of the study population according to the severity of acute pancreatitis

	Mild	Moderate	Severe	*P* ^‡^
	(*n* = 156)	(*n* = 57)	(*n* = 23)	
Age (years)^*^	56 (42–68)	52 (40–67)	62 (52–77)	0·064
Sex ratio (F : M)	91 : 65	22 : 35	11 : 12	0·040^§^
Updated Charlson co-morbidity index score^*^	0 (0–1)	0 (0–1)	0 (0–1)	0·158
Aetiology				
Biliary	83 (53·2)	29 (51)	8 (35)	0·256
Alcohol	30 (19·2)	13 (23)	5 (22)	0·835
Other	43 (27·6)	15 (26)	10 (43)	0·259
Time to admission (h)^*^	8 (4–14)	6 (4–12)	11 (6–15)	0·111
Time from admission to sampling (h)^*^	14 (8–20)	17 (10–20)	16 (12–20)	0·588
Worst modified CT severity index^*^^†^	2 (0–2)	6 (4–8)	6 (6–8)	< 0·001^§^
Acute peripancreatic fluid collection	0 (0)	23 (40)	7 (30)	< 0·001^§^
Pancreatic necrosis	0 (0)	19 (33)	11 (48)	< 0·001^§^
Infected pancreatic necrosis and/or sepsis	0 (0)	1 (2)	8 (35)	< 0·001^¶^
Need for ICU admission	0 (0)	0 (0)	19 (83)	< 0·001^¶^
Need for antibiotics	9 (5·8)	9 (16)	16 (70)	< 0·001^#^
Nutritional support	0 (0)	0 (0)	13 (57)	< 0·001^¶^
Necrosectomy and/or percutaneous drainage	0 (0)	3 (5)	8 (35)	< 0·001^#^
Death	0 (0)	0 (0)	9 (39)	< 0·001^¶^
Length of hospital stay (days)^*^	5·5 (3–9)	14 (11–21)	29 (13·5–65·5)	< 0·001^#^

Values in parentheses are percentages unless indicated otherwise;

* values are median (i.q.r.).

† CT performed in 31 patients with mild, 48 with moderate and 20 with severe pancreatitis.

‡ χ^2^ or Fisher's exact test for categorical data and Kruskal–Wallis test for continuous data.

§ *P* < 0·050, mild *versus* moderate or severe;

¶ *P* < 0·050, severe *versus* mild or moderate;

# *P* < 0·050 between any two groups (χ^2^ or Fisher's exact test for categorical data and Mann–Whitney *U* test for continuous data).

### Prediction of disease severity

**Fig. 3:**
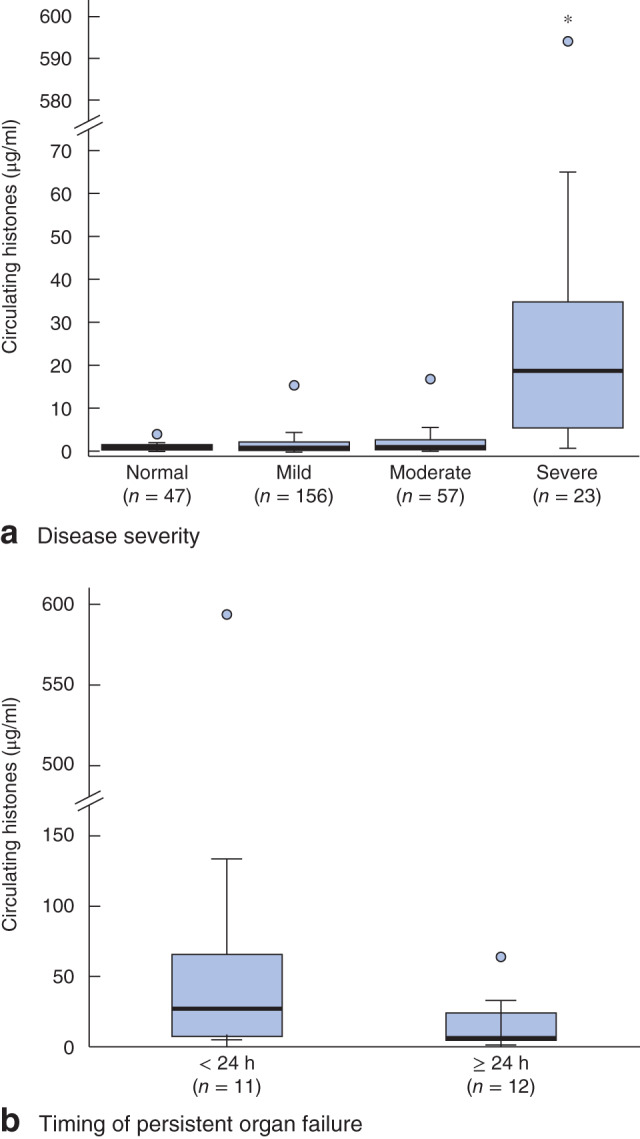
Comparison of circulating histone levels measured within 24 h of admission: **a** healthy volunteers and patients with acute pancreatitis on admission; **b** patients with persistent organ failure occurring in less than 24 h *versus* 24 h or more. Median values (bold line), i.q.r. (box), and range (error bars) including outliers (circles) are shown. *P < 0·050 *versus* each other group (Mann–Whitney *U* test)

Levels of circulating histones in healthy volunteers and patients with acute pancreatitis are shown in *Fig. 3a*. Representative western blots for histone measurement are available in *Fig. S2* (supporting information). Circulating histones were barely detectable in healthy volunteers, and comparable in patients with mild and moderate disease (median (i.q.r.) 1·1 (0·6–2·1) *versus* 1·3 (0·5–2·8) μg/ml; *P =* 0·633) (*Table 2*). Circulating histone levels were raised significantly only in patients with severe disease (18·8 (5·9–33·8) μg/ml; *P* < 0·001 *versus* all other groups), and were higher when persistent organ failure occurred within 24 h than more than 24 h after admission (*P* = 0·056) (*Fig. 3b*).

**Table 2:** Comparison of clinical scores and biomarkers in different severity groups

	Mild	Moderate	Severe	*P* ^*^
	(*n* = 156)	(*n* = 57)	(*n* = 23)	
Clinical scores within 24 h of admission				
SIRS	1 (0–1)	1 (1–2)	2 (1–2)	0·002^†^
BISAP	1 (0–1)	1 (0–1)	2 (1–2)	< 0·001^‡^
APACHE II	5 (3–7)	7 (5–9)	10 (6–12·5)	< 0·001^†^
SOFA	0 (0–1)	1 (0–2)	2 (1–4)	< 0·001^†^
Biomarkers within 24 h of admission				
White cell count (×10^9^/l)	12·4 (9·9–15·3)	14·2 (11·5–17·4)	14·4 (11·2–19·3)	0·002^‡^
Neutrophil to lymphocyte ratio	6·9 (4·1–14·8)	8 (4·3–14·7)	7·7 (5·3–23·6)	0·520
Haematocrit (%)	40·1 (38–42·9)	43·5 (39·3–45·6)	42·8 (37·6–45·4)	0·003^‡^
Urea (mmol/l)	5 (3·7–6·1)	4·8 (3·7–6·3)	7·3 (5·2–8·9)	0·001^§^
Creatinine (μmol/l)	71 (61–88)	83 (65–99)	104 (75–157·5)	< 0·001^†^
CRP (mg/l)	8 (5–23)	10 (5–34)	10 (5–137)	0·288
IL-6 (pg/ml)	13·8 (7–57·8)	30·8 (8·6–96·2)	65·1 (21·7–143·4)	0·022^¶^
IL-8 (pg/ml)	9·9 (0·4–19·1)	13·9 (5·4–28·9)	44·5 (18·8–64·2)	< 0·001^¶^
Circulating histones (μg/ml)	1·1 (0·6–2·1)	1·3 (0·5–2·8)	18·8 (5·9–33·8)	< 0·001^§^
Biomarkers at 48 h after admission				
Urea (mmol/l)	3·5 (2·6–4·7)	3·5 (2·7–5·5)	8·7 (5·2–11·7)	< 0·001^§^
Creatinine (μmol/l)	66 (53·5–79)	65 (54·5–86)	71 (57–172)	0·080
CRP (mg/l)	38 (11–116)	234 (159–317)	328 (250–368)	0·001^†^

Values are median (i.q.r.). SIRS, Systemic Inflammatory Response Syndrome; BISAP, Bedside Index for Severity in Acute Pancreatitis; APACHE, Acute Physiology And Chronic Health Evaluation; SOFA, Sequential Organ Failure Assessment; CRP, C-reactive protein; IL, interleukin.

* Kruskal–Wallis test.

† *P* < 0·050 between any two groups;

‡ *P* < 0·050, mild *versus* moderate or severe

§ *P* < 0·050, severe *versus* mild or moderate;

¶ *P* < 0·050, mild *versus* severe (Mann–Whitney *U* test).

The levels of circulating histones and other clinical parameters used for assessing acute pancreatitis severity are summarized in *Table 2*. Like circulating histones, SIRS, BISAP, APACHE II and SOFA scores all increased with disease severity within 24 h of admission, and were significantly higher in those with severe disease compared with the mild or moderate groups. Levels of urea, creatinine, haematocrit, IL-6 and IL-8 were also significantly higher in patients with severe disease. In contrast, CRP levels showed no significant association with disease severity within 24 h of admission, but became significant after 48 h. These data indicate that, following the onset of acute pancreatitis, histones appear within the circulation more rapidly than CRP and synchronously with severe clinical manifestations, and may have potential value in the early prediction of severe acute pancreatitis.

### Prediction of persistent organ failure

**Fig. 4:**
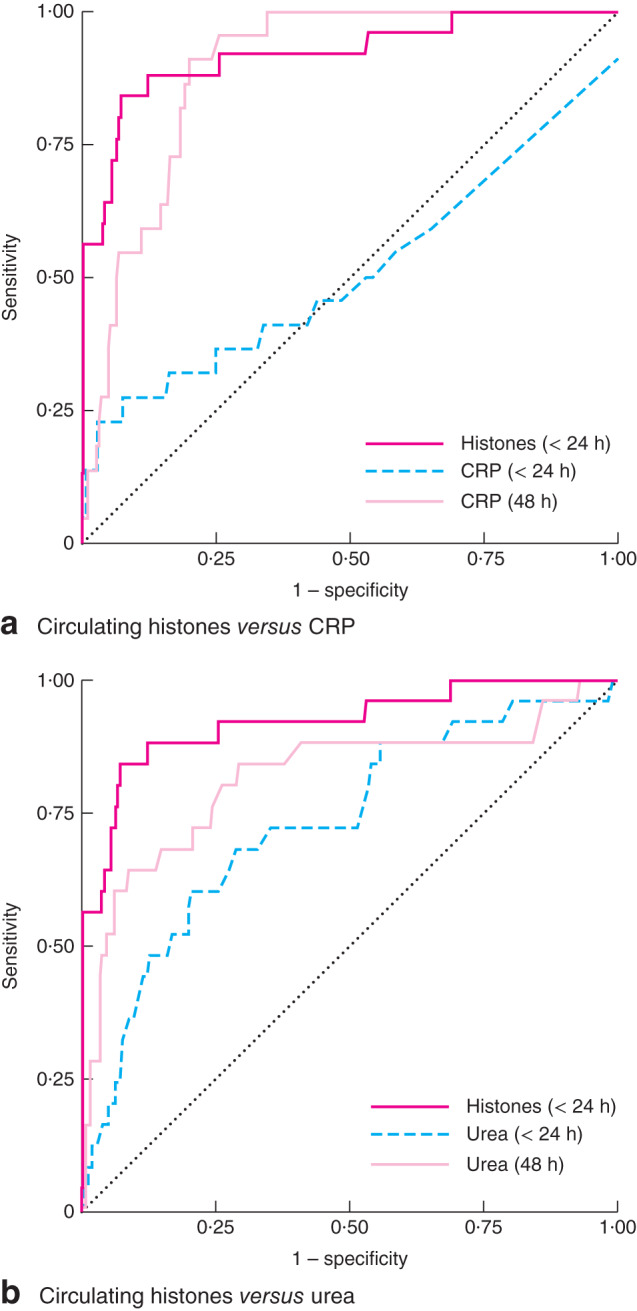
Comparison of receiver operating characteristic (ROC) curves for prediction of persistent organ failure since admission: **a** circulating histones within 24 h *versus* C-reactive protein (CRP) within 24 h or at 48 h; **b** circulating histones within 24 h *versus* urea within 24 h or at 48 h. Dotted line is the ROC reference line

The AUC values for potential predictors of persistent organ failure are summarized in *Table 3*. The values for all the clinical scores were moderate (range 0·68–0·81) in predicting persistent organ failure, and both the sensitivity (51·6–68·4 per cent) and specificity (74·1–82·8 per cent) were poor. Levels of circulating histones within 24 h of admission outperformed clinical scores and had stronger predictive value (AUC 0·92, 95 per cent c.i. 0·85 to 0·99) than either CRP (AUC 0·54, 0·37 to 0·70) (*Fig. 4a*) or urea (AUC 0·75, 0·63 to 0·86) (*Fig. 4b*). CRP (AUC 0·89, 0·84 to 0·94) (*Fig. 4a*) and urea (AUC 0·82, 0·71 to 0·94) (*Fig. 4b*) only showed relatively strong predictive value for persistent organ failure at 48 h following admission. Using the optimal cut-off value of circulating histones (5·4 μg/ml), they surpassed all other parameters measured in this study, with a sensitivity, specificity, PPV and NPV of 82·6, 94·4, 61·3 and 98·1 per cent respectively, a PLR and NLR of 14·7 and 0·18, and post-test probability of 61·4 per cent (*versus* prevalence 9·7 per cent) (*Table 4*). However, combining circulating histone levels with either CRP or urea at 48 h did not increase the predictive values further.

**Table 3:** Accuracy of potential predictors with time since admission

	Persistent organ failure		Mortality	
	AUC	*P*	AUC	*P*
Clinical scores within 24 h of admission				
SIRS	0·68 (0·55, 0·81)	0·010	0·72 (0·53, 0·91)	0·037
BISAP	0·81 (0·71, 0·91)	< 0·001	0·90 (0·80, 0·99)	< 0·001
APACHE II	0·74 (0·62, 0·87)	< 0·001	0·86 (0·70, 1·00)	0·001
SOFA	0·79 (0·68, 0·90)	< 0·001	0·83 (0·66, 0·99)	0·001
Biomarkers within 24 h of admission				
White cell count (×10^9^/l)	0·62 (0·49, 0·74)	0·066	0·67 (0·46, 0·87)	0·085
Haematocrit (%)	0·58 (0·43, 0·73)	0·253	0·52 (0·30, 0·74)	0·848
Urea (mmol/l)	0·75 (0·63, 0·86)	< 0·001	0·83 (0·69, 0·98)	0·001
Creatinine (μmol/l)	0·74 (0·62, 0·86)	< 0·001	0·91 (0·81, 1·00)	< 0·001
CRP (mg/l)	0·54 (0·37, 0·70)	0·612	0·75 (0·54, 0·96)	0·026
IL-6 (pg/ml)	0·67 (0·49, 0·74)	0·018	0·73 (0·54, 0·91)	0·045
IL-8 (pg/ml)	0·76 (0·64, 0·89)	0·001	0·89 (0·78, 0·99)	0·001
Circulating histones (μg/ml)	0·92 (0·85, 0·99)	< 0·001	0·96 (0·92, 1·00)	< 0·001
Biomarkers at 48 h after admission				
Urea (mmol/l)	0·82 (0·71, 0·94)	< 0·001	0·97 (0·95, 0·99)	< 0·001
Creatinine (μmol/l)	0·61 (0·44, 0·78)	0·129	0·86 (0·65, 1·00)	0·002
CRP (mg/l)	0·89 (0·84, 0·94)	< 0·001	0·86 (0·79, 0·93)	0·003

Values in parentheses are 95 per cent confidence intervals. AUC, area under the receiver operating characteristic (ROC) curve; SIRS, Systemic Inflammatory Response Syndrome; BISAP, Bedside Index for Severity in Acute Pancreatitis; APACHE, Acute Physiology And Chronic Health Evaluation; SOFA, Sequential Organ Failure Assessment; CRP, C-reactive protein; IL, interleukin.

**Table 4:** Predictive values of the most effective predictors since admission

	Cut-off value	Sensitivity (%)	Specificity (%)	PPV (%)	NPV (%)	PLR	NLR	PP (%)
Persistent organ failure (prevalence 9·7%)								
BISAP (< 24 h)	≥ 2	68·4	82·8	26·5	96·7	4·0	0·38	30·2
Circulating histones (< 24 h)	≥ 5·4 μg/ml	82·6	94·4	61·3	98·1	14·7	0·18	61·4
Urea (48 h)	≥ 8 mmol/l	60·9	94·7	56·0	95·6	11·5	0·41	55·4
CRP (48 h)	≥ 250 mg/l	80·0	80·5	29·1	97·6	4·1	0·25	30·7
Mortality (prevalence 3·8%)								
BISAP (< 24 h)	≥ 2	87·5	80·9	14·3	99·4	4·6	0·15	15·4
Circulating histones (< 24 h)	≥ 5·4 μg/ml	88·9	89·9	25·8	99·5	8·8	0·12	25·9
Urea (48 h)	≥ 8 mmol/l	100	93·2	37·5	100	14·8	0	37·0
CRP (48 h)	≥ 250 mg/l	100	77·1	10·9	100	4·4	0	14·9

PPV, positive predictive value; NPV, negative predictive value; PLR, positive likelihood ratio; NLR, negative likelihood ratio; PP, post-test probability based on the test result being above the cut-off value; BISAP, Bedside Index for Severity in Acute Pancreatitis; CRP, C-reactive protein.

### Prediction of major infection

For predicting major infection, the AUC for circulating histones was 0·78 (0·62 to 0·94), which was similar to, or lower than the values for BISAP, APACHE II, SOFA, urea, creatinine and IL-6 (range 0·80 to 0·87) within 24 h (*Table S1*, supporting information). At 48 h, the AUC values for CRP (*Fig. S3*a, supporting information) and urea (*Fig. S3*b, supporting information) were 0·83 and 0·92 respectively, higher than that for circulating histones within 24 h. At a cut-off of 5·4 μg/ml, the sensitivity, specificity, PPV and NPV of circulating histones for the prediction of major infection were 44·4, 88·1, 12·9 and 97·6 per cent respectively, with a PLR and NLR of 3·7 and 0·63, and post-test probability of 12·8 per cent (*versus* prevalence 3·8 per cent) (*Table S2*, supporting information).

### Prediction of mortality

Circulating histones had a higher predictive value for death (AUC 0·96, 95 per cent c.i. 0·92 to 1·00) than any other parameter within 24 h (*Table 3*), including both CRP (*Fig. S4*a, supporting information) and urea (*Fig. S4*b, supporting information), but was comparable to urea at 48 h (AUC 0·97, 0·95 to 0·99) (*Table 3*). At an optimal cut-off of 5·4 μg/ml, the sensitivity, specificity, PPV and NPV of circulating histones for predicting death were 88·9, 89·9, 25·8 and 99·5 respectively, with a PLR and NLR 8·8 and 0·12, and a post-test probability of 25·9 per cent (*versus* prevalence 3·8 per cent) (*Table 4*). For the urea level at 48 h with a cut-off of 8 mmol/l, the sensitivity, specificity, PPV and NPV were 100, 93·2, 37·5 and 100 per cent, with a PLR and NLR of 14·8 and 0, and a post-test probability of 37·0 per cent (*Table 4*). Both BISAP within 24 h (cut-off 2) and CRP (cut-off 250 mg/l) at 48 h had reasonable predictive values, but PLR values (4·6 and 4·4 respectively) were much lower than those of both circulating histones within 24 h and urea at 48 h. Combining circulating histones (cut-off 5·4 μg/ml) with either urea (cut-off 8 mmol/l, at 48 h) or CRP (cut-off 250 mg/l, at 48 h) did not increase specificity compared with the individual parameters alone.

### Leucocyte viability, local complications and transient organ failure

Circulating levels of histones have been shown to correlate significantly with pancreatic necrosis scores in animal models^16^. However, the correlation between histone levels on admission and the first (*n* = 99; *r* = 0·17, *P* = 0·094) or the worst (*n* = 99; *r* = 0·195, *P* = 0·054) MCTSI value were not significant. In an analysis including only patients with mild and moderate disease, there was no correlation between circulating histone levels and local complications, acute peripancreatic fluid collection, pancreatic necrosis or transient organ failure with or without local complications (all *P* ≥ 0·237). During disease progression, pancreatic necrosis normally occurs 24 h after onset of symptoms and would therefore not directly affect histone levels within the first 24 h, but may be contributory after this time. Another source could be histones released from immune cells following cellular damage or death. The percentage of viable leucocytes was measured in the peripheral blood of 62 patients in this cohort, and a significant negative association was found between circulating histone levels and leucocyte viability within 24 h of admission (*r* = –0·511, *P* = 0·001). There was no significant impact of aetiology (biliary, alcohol or others) on circulating histone levels (*r* = –0·024, *P* = 0·712) or leucocyte viability (*r* = –0·101, *P* = 0·426).

## Discussion

In this study of consecutive patients with acute pancreatitis, levels of circulating histones were an accurate index of disease severity, and capable of predicting persistent organ failure and mortality; they performed better than BISAP and urea, indices currently used in the clinical setting within 24 h of admission (48 h from disease onset to admission).

Both revised Atlanta^2^ and determinant-based^3^ classifications for severity stratification of acute pancreatitis recognize persistent organ failure as the predominant determinant of death. The present findings confirm this and mortality occurred only in the group with severe disease. Circulating histones had the best AUC value in predicting both persistent organ failure and mortality within 24 h of admission. On the other hand, patients with transient organ failure without local complications have a similar clinical outlook to those with mild disease^31^. Current clinical scores are imprecise at differentiating between organ failure that will be transient or persistent^32^. In the present cohort, only 11 of 23 patients with severe acute pancreatitis developed persistent organ failure within 24 h of admission, but circulating histone levels were still invaluable in differentiating this group from patients with transient organ failure. Only one patient with transient organ failure had circulating histone levels that exceeded the cut-off value for predicting persistent organ failure.

This study showed that circulating histones have moderate predictive value for major infection, underlining the challenges in fulfilling the clinical need in this area in the current absence of a good predictor^10^. Here, five of nine patients died within the first week of admission, precluding further assessment of the development of infected pancreatic necrosis. This introduces bias for circulating histones in predicting major infection. Patients with symptomatic sterile necrosis invariably develop infection after surgical intervention^33,34^. A previous study^5^ showed that baseline parameters and clinical outcomes were similar in patients with a primary diagnosis of infected pancreatic necrosis and those who presented initially with sterile necrosis but eventually required surgery despite maximal conservative treatment^5^. Infected pancreatic necrosis has been emphasized by the determinant-based classification as another determinant of mortality^3,4^. The modified determinant-based classification^35^ further stratifies patients with persistent organ failure into two groups: one with infected pancreatic necrosis and one without. It would be of interest to test whether circulating histone levels differ between these two groups in a large study.

Strong correlations have been observed between peak histone levels and pancreatic necrosis in animal models of acute pancreatitis^16^, but histone levels within 24 h of admission in this study did not correlate with MCTSI or predict pancreatic necrosis. This discrepancy may be due to the time point of blood collection or a fundamental difference in disease progression, as pancreatic necrosis occurs several days later in humans than in animal models. Therefore, pancreatic acinar cells are likely to make a limited contribution to levels of circulating histones in early-stage disease. Instead, immune cells such as neutrophils may be a major contributor to histone release, either via neutrophil extracellular trap formation (NETosis)^19^ or necrosis. The present observation that histone levels significantly and inversely correlated with the proportion of dead or dying peripheral leucocytes supports this argument. More data are needed to establish this point. The authors postulate that histone levels may reflect the intensity of systemic inflammation.

The most studied DAMP in the acute pancreatitis setting is HMGB1^12^. Based on previous reports^13^, significant release of HMGB1 occurs beyond 24 h in experimental acute pancreatitis, which implies that it may not be useful in the early prediction of persistent organ failure. In contrast, the authors^16^ have previously demonstrated that levels of circulating histones rise within 2 h in experimental and early in human acute pancreatitis, as demonstrated here. Circulating nucleosomes have been tested in non-consecutive patients with acute pancreatitis based on the revised Atlanta classification^17^, but the predictive values were lower than those in the present study. This discrepancy may be explained by the different pathophysiological roles of circulating nucleosomes and histones. One of the fundamental differences is that nucleosomes are not toxic when released in an intact form^36^. Current assays cannot distinguish between intact and degraded nucleosomes, which feeds into the controversy surrounding their clinical value compared with the well established toxic effects of circulating histones^37^. As the present study included only 23 patients with severe acute pancreatitis, larger studies are needed to compare the predictive values of these nuclear DAMPs with current clinical indices in the setting of acute pancreatitis. Further studies are also needed to elucidate the temporal changes and source of circulating histones in acute pancreatitis, and how these correlate with systemic inflammation markers and individual organ failure. Such data could advance the consideration of monitoring histones routinely in clinical practice as well as the translational potential of targeting circulating histones in acute pancreatitis.

## Supplementary Material

bjs10538-sup-0001-Figures **Fig. S1** Timing of persistent organ failure after admission. D, day
**Fig. S2** Representative western blots for circulating histone measurement. Recombinant histone H3 was used as standard, and specific antihistone 3 antibody was used to measure histones. Normal, heathy volunteers; Se, severe pancreatitis; Mi, mild pancreatitis; Mo, moderate pancreatitis
**Fig. S3** Comparison of receiver operating characteristic (ROC) curves for prediction of major infection since admission: **a** circulating histones within 24 h *versus* C-reactive protein (CRP) within 24 h or at 48 h; **b** circulating histones within 24 h *versus* urea within 24 h or at 48 h. Dashed line is the ROC reference line
**Fig. S4** Comparison of receiver operating characteristic (ROC) curves for prediction of mortality since admission: **a** circulating histones within 24 h *versus* C-reactive protein (CRP) within 24 h or at 48 h; **b** circulating histones within 24 h *versus* urea within 24 h or at 48 h. Dashed line is the ROC reference line

bjs10538-sup-0002-Tables **Table S1** Accuracy of potential predictors of major infection with time since admission
**Table S2** Comparison of predictive values of the most effective predictors for major infection since admission
