# Supplementary material for: Accuracy of circulating histones in predicting persistent organ failure and mortality in patients with acute pancreatitis
Source: Br J Surg. 2017 Apr 24;104(9):1215–25. doi: 10.1002/bjs.10538 (PMC7938821; doi:10.1002/bjs.10538)
Supplement: bjs10538-sup-0001-Figures — Fig. S1 Timing of persistent organ failure after admission. D, day Fig. S2 Representative western blots for circulating histone measurement. Recombinant histone H3 was used as standard, and specific antihistone 3 antibody was used to measure histones. Normal, heathy volunteers; Se, severe pancreatitis; Mi, mild pancreatitis; Mo, moderate pancreatitis Fig. S3 Comparison of receiver operating characteristic (ROC) curves for prediction of major infection since admission: a circulating histones within 24 h versus C-reactive protein (CRP) within 24 h or at 48 h; b circulating histones within 24 h versus urea within 24 h or at 48 h. Dashed line is the ROC reference line Fig. S4 Comparison of receiver operating characteristic (ROC) curves for prediction of mortality since admission: a circulating histones within 24 h versus C-reactive protein (CRP) within 24 h or at 48 h; b circulating histones within 24 h versus urea within 24 h or at 48 h. Dashed line is the ROC reference line [file bjs10538-sup-0001-figures.pptx]

## Slide 1
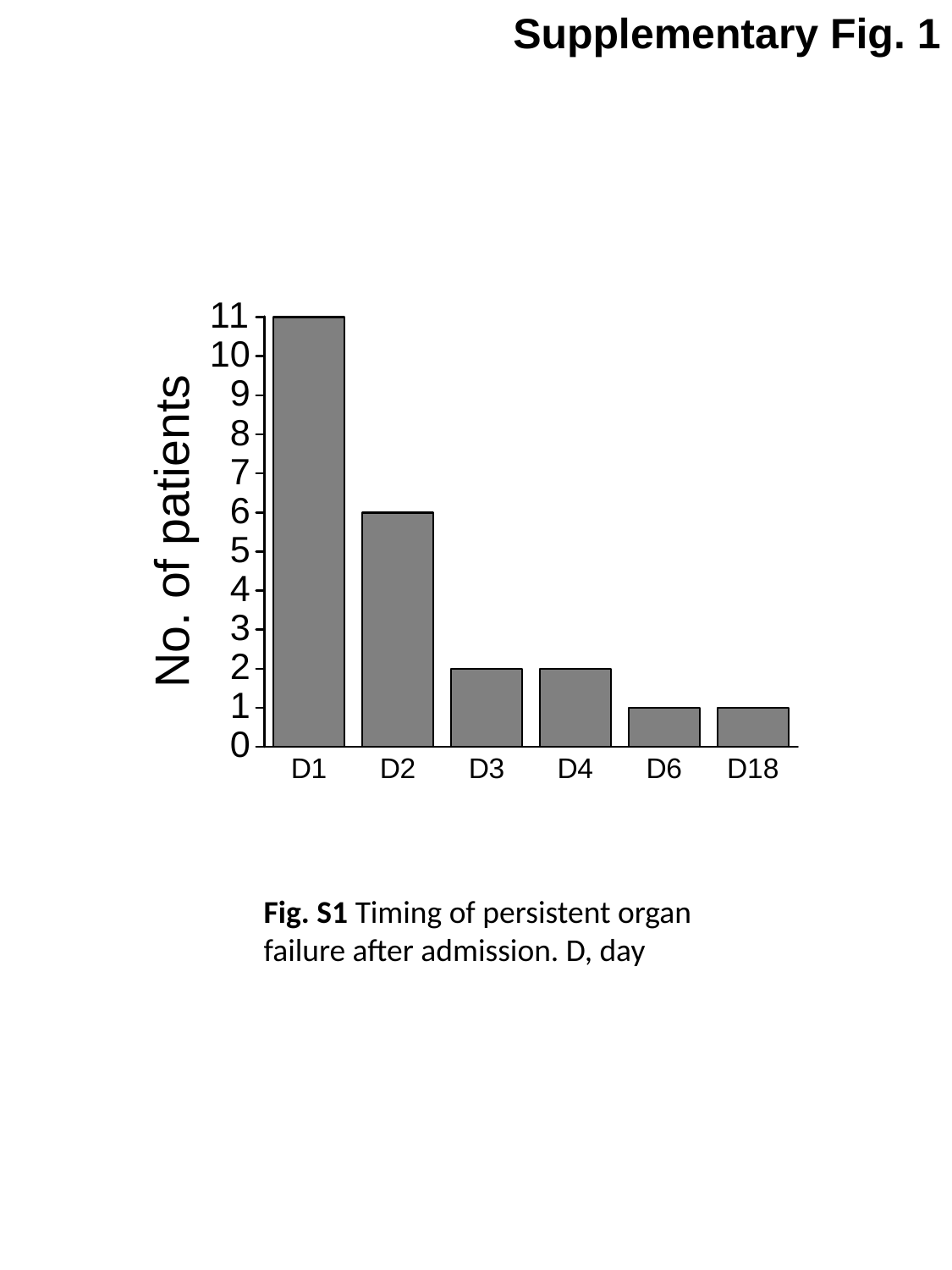

Supplementary Fig. 1
Fig. S1 Timing of persistent organ failure after admission. D, day

## Slide 2
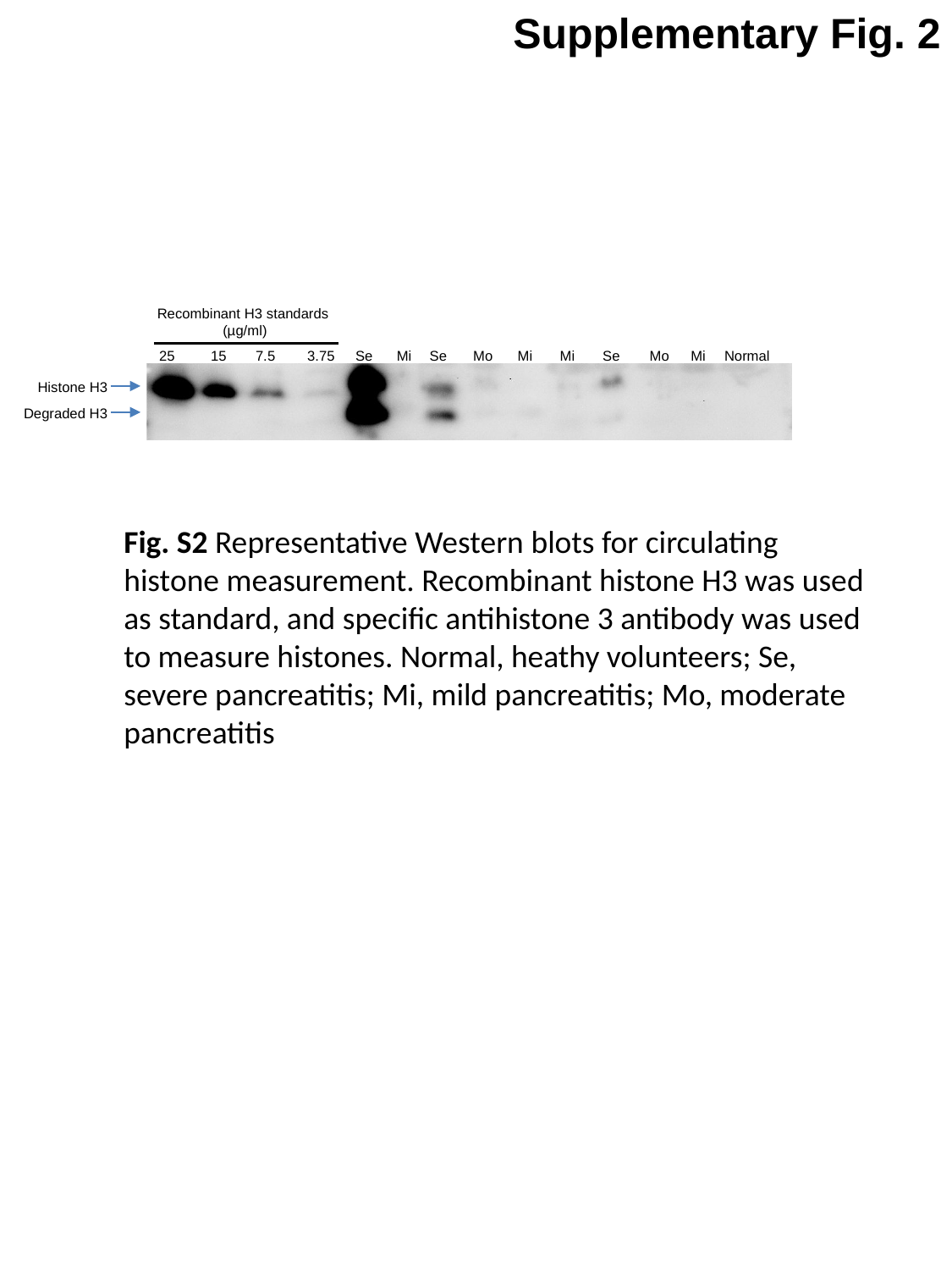

Supplementary Fig. 2
Recombinant H3 standards
 (µg/ml)
25
15
7.5
3.75
Se
Mi
Se
Mo
Mi
Mi
Se
Mo
Mi
Normal
Histone H3
Degraded H3
Fig. S2 Representative Western blots for circulating histone measurement. Recombinant histone H3 was used as standard, and specific antihistone 3 antibody was used to measure histones. Normal, heathy volunteers; Se, severe pancreatitis; Mi, mild pancreatitis; Mo, moderate pancreatitis

## Slide 3
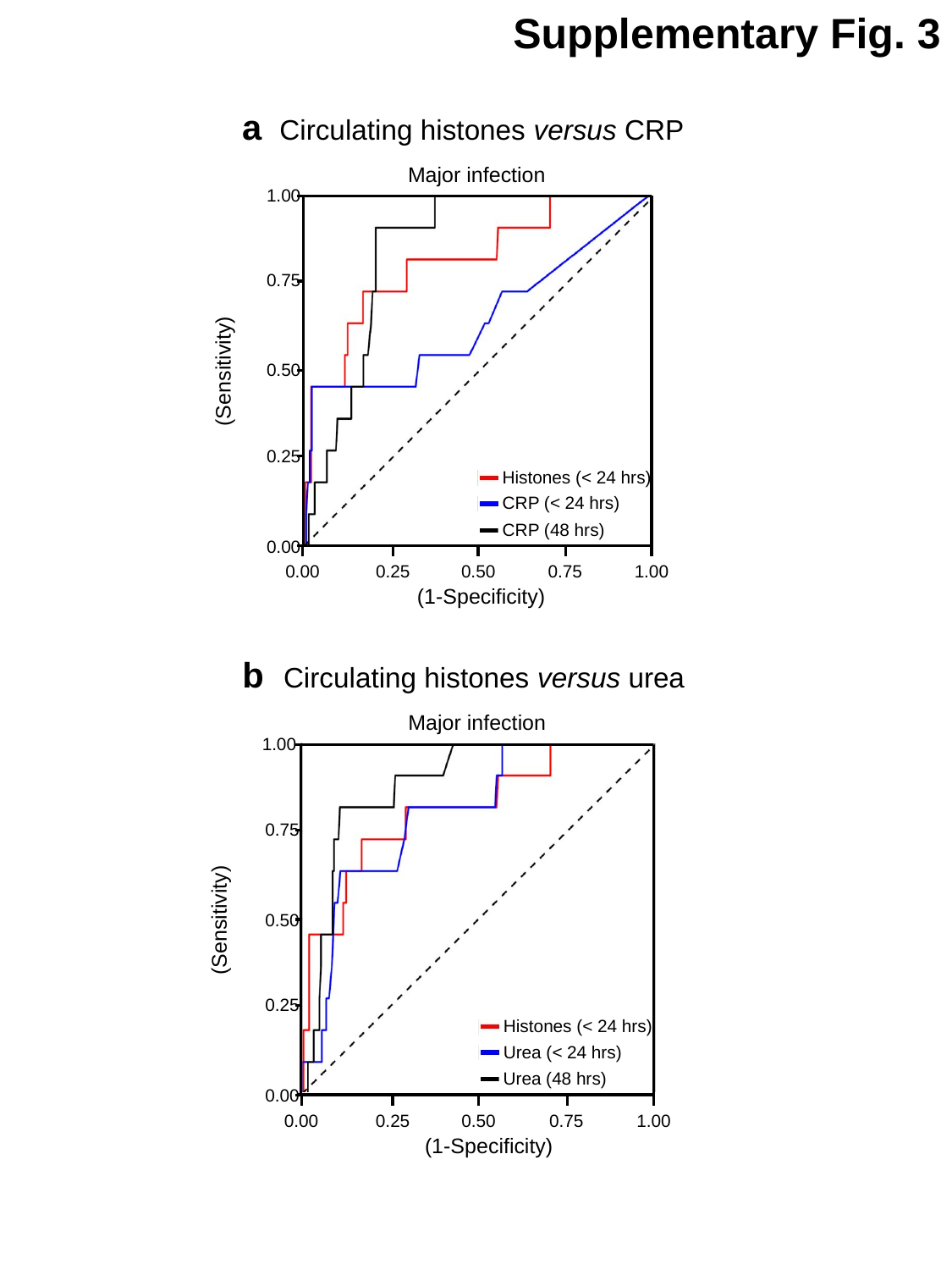

Supplementary Fig. 3
a Circulating histones versus CRP
Major infection
1.00
0.75
(Sensitivity)
0.50
0.25
Histones (< 24 hrs)
CRP (< 24 hrs)
CRP (48 hrs)
0.00
0.00
0.25
0.50
0.75
1.00
 (1-Specificity)
b Circulating histones versus urea
Major infection
1.00
0.75
(Sensitivity)
0.50
0.25
Histones (< 24 hrs)
Urea (< 24 hrs)
Urea (48 hrs)
0.00
0.00
0.25
0.50
0.75
1.00
 (1-Specificity)

## Slide 4
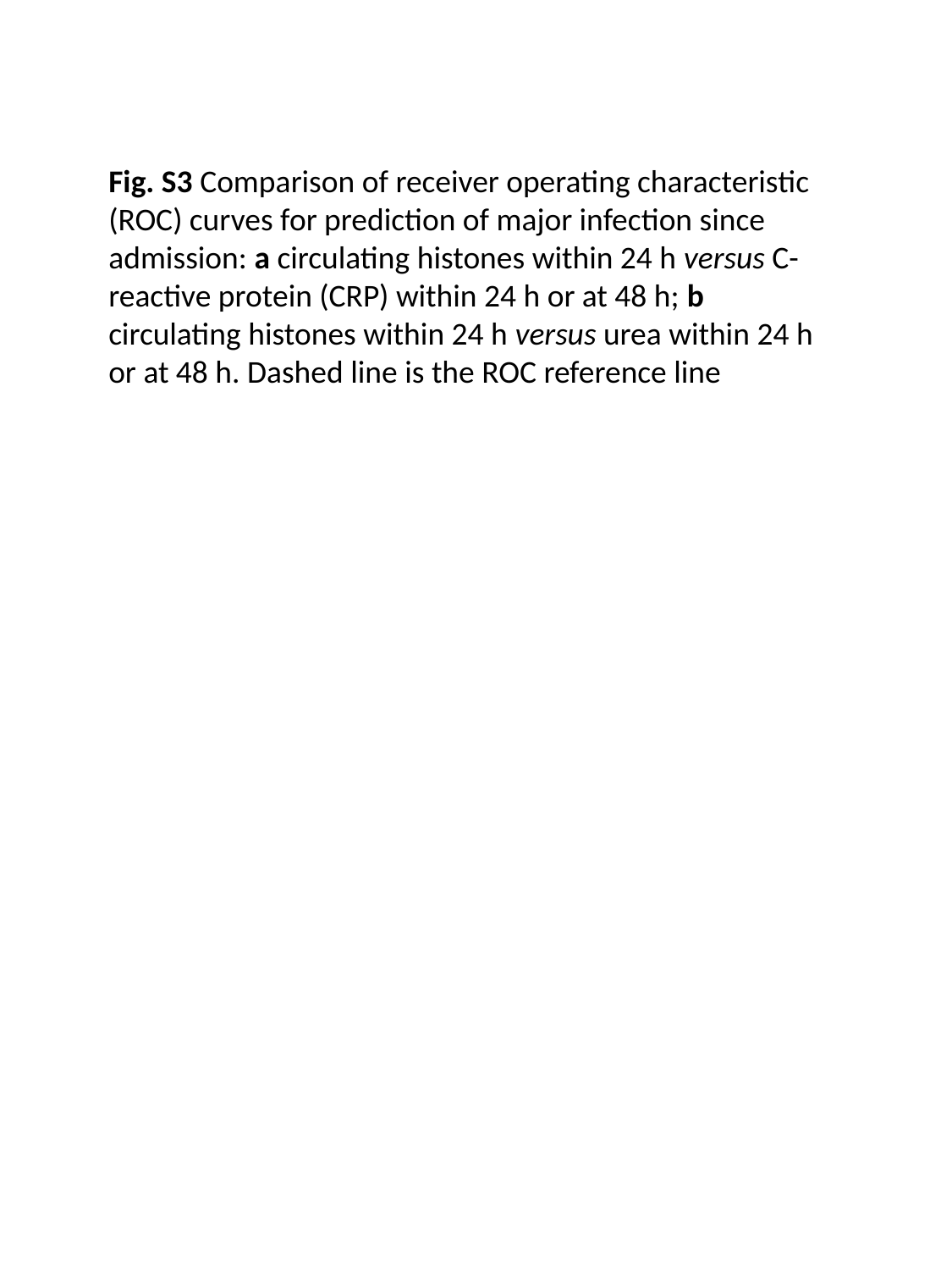

Fig. S3 Comparison of receiver operating characteristic (ROC) curves for prediction of major infection since admission: a circulating histones within 24 h versus C-reactive protein (CRP) within 24 h or at 48 h; b circulating histones within 24 h versus urea within 24 h or at 48 h. Dashed line is the ROC reference line

## Slide 5
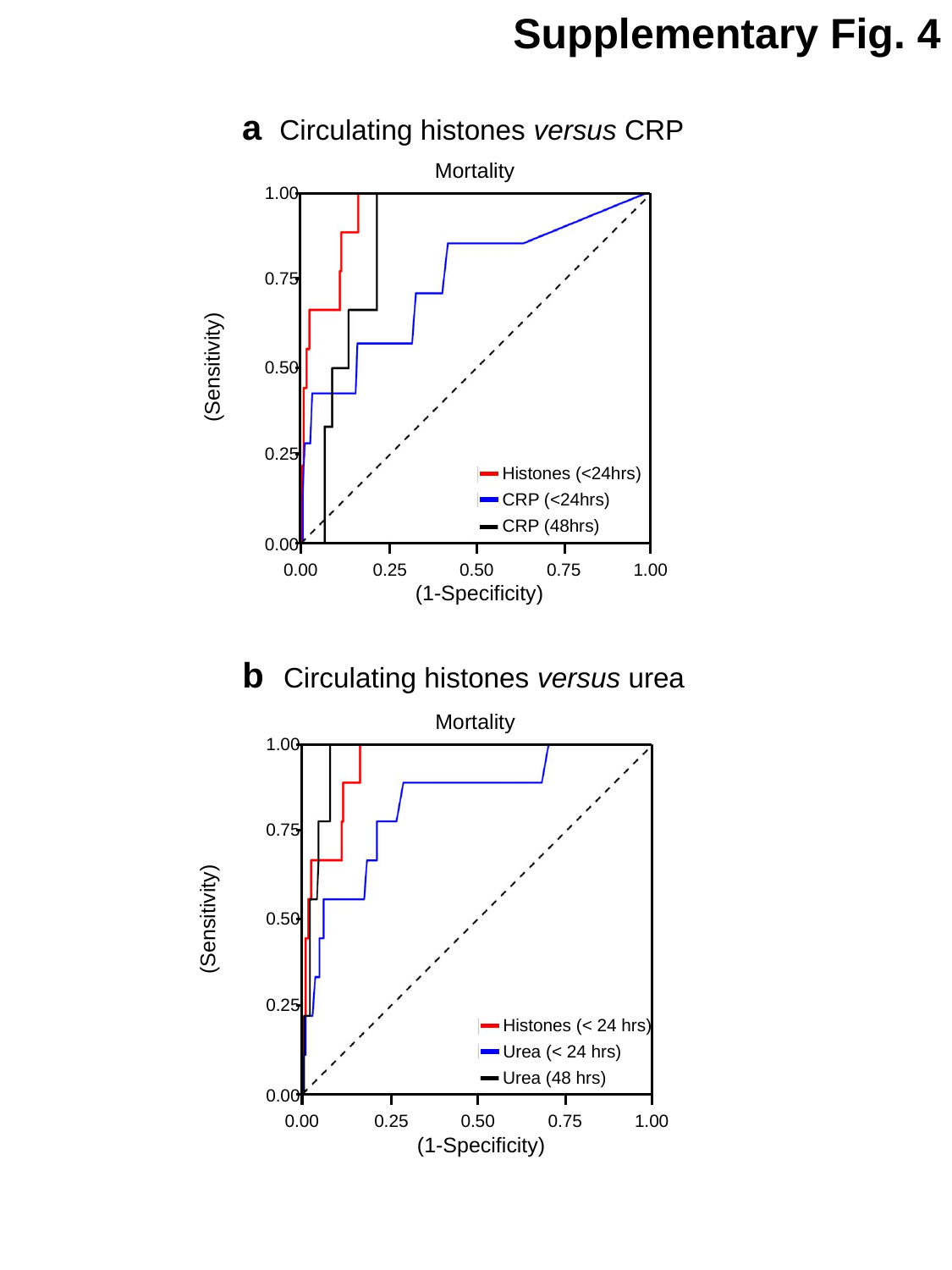

Supplementary Fig. 4
a Circulating histones versus CRP
Mortality
1.00
0.75
(Sensitivity)
0.50
0.25
Histones (<24hrs)
CRP (<24hrs)
CRP (48hrs)
0.00
0.00
0.25
0.50
0.75
1.00
 (1-Specificity)
b Circulating histones versus urea
Mortality
1.00
0.75
(Sensitivity)
0.50
0.25
Histones (< 24 hrs)
Urea (< 24 hrs)
Urea (48 hrs)
0.00
0.00
0.25
0.50
0.75
1.00
 (1-Specificity)

## Slide 6
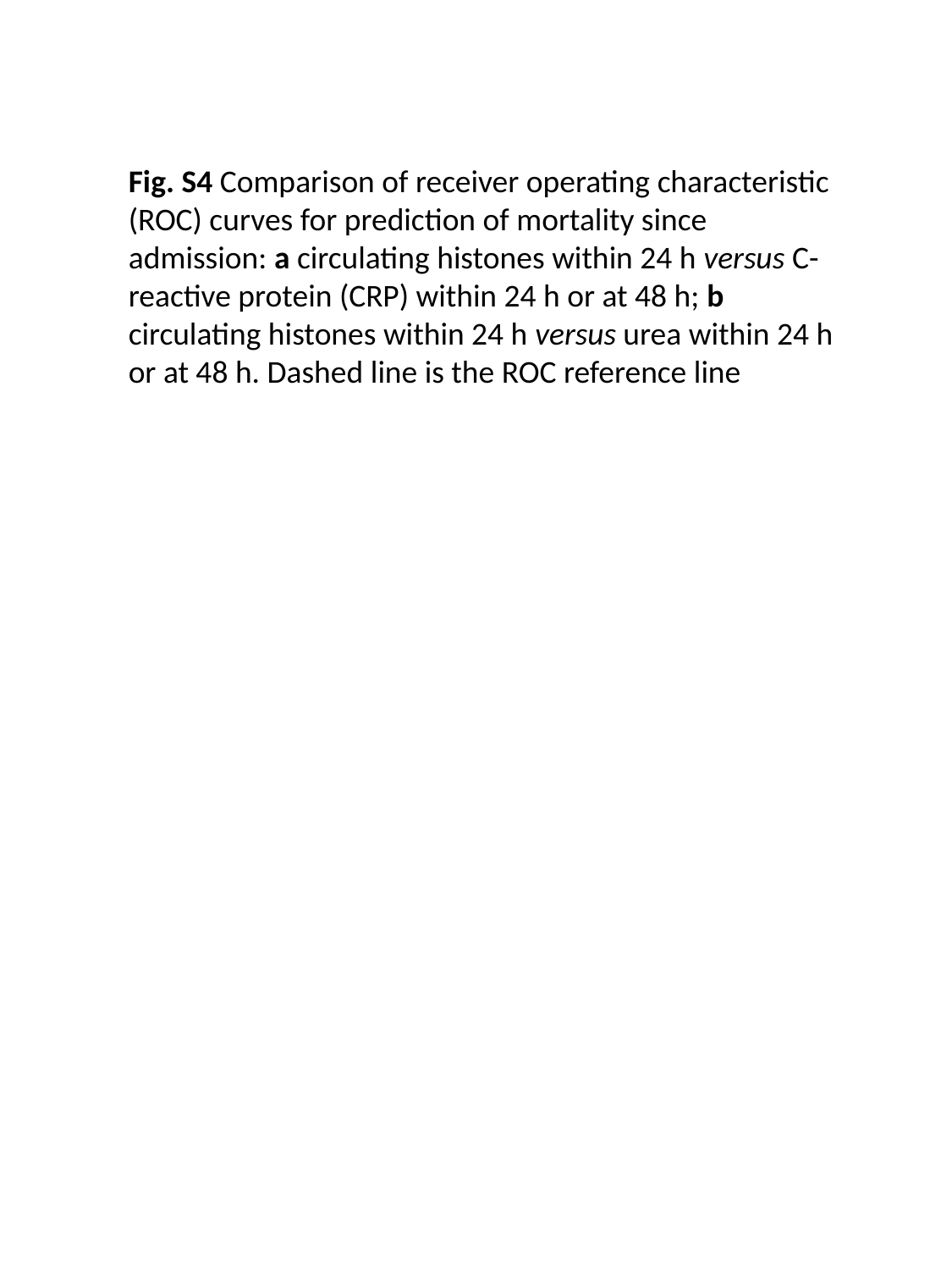

Fig. S4 Comparison of receiver operating characteristic (ROC) curves for prediction of mortality since admission: a circulating histones within 24 h versus C-reactive protein (CRP) within 24 h or at 48 h; b circulating histones within 24 h versus urea within 24 h or at 48 h. Dashed line is the ROC reference line
