# Supplementary material for: Accuracy of circulating histones in predicting persistent organ failure and mortality in patients with acute pancreatitis
Source: Br J Surg. 2017 Apr 24;104(9):1215–25. doi: 10.1002/bjs.10538 (PMC7938821; doi:10.1002/bjs.10538)
Supplement: bjs10538-sup-0002-Tables — Table S1 Accuracy of potential predictors of major infection with time since admission Table S2 Comparison of predictive values of the most effective predictors for major infection since admission [file bjs10538-sup-0002-tables.docx]

**BJS10538**

**Accuracy of circulating histones in predicting persistent organ failure and mortality in patients with acute pancreatitis**

T. Liu, W. Huang, P. Szatmary, S. T. Abrams, Y. Alhamdi, Z. Lin, W. Greenhalf, G. Wang, R. Sutton and C. H. Toh

**Table S1** Accuracy of potential predictors of major infection with time since admission

|  | AUC | *P* |
| --- | --- | --- |
| Clinical scores within 24 h of admission |  |  |
| SIRS | 0.67 (0.48, 0.87) | 0.096 |
| BISAP | 0.83 (0.72, 0.94) | 0.002 |
| APACHE II | 0.87 (0.75, 1.00) | < 0.001 |
| SOFA | 0.82 (0.64, 0.99) | 0.001 |
| Biomarkers within 24 h of admission |  |  |
| White cell count (×10^9^/l) | 0.67 (0.47, 0.86) | 0.087 |
| Haematocrit (%) | 0.60 (0.35, 0.86) | 0.316 |
| Urea (mmol/l) | 0.86 (0.74, 0.97) | < 0.001 |
| Creatinine (μmol/l) | 0.83 (0.71, 0.96) | 0.001 |
| CRP (mg/l) | 0.72 (0.50, 0.94) | 0.026 |
| IL-6 (pg/ml) | 0.73 (0.57, 0.87) | 0.031 |
| IL-8 (pg/ml) | 0.80 (0.67, 0.94) | 0.005 |
| Circulating histones (μg/ml) | 0.78 (0.62, 0.94) | 0.005 |
| Biomarkers at 48 h after admission |  |  |
| Urea (mmol/l) | 0.92 (0.86, 0.97) | < 0.001 |
| Creatinine (μmol/l) | 0.62 (0.36, 0.88) | 0.253 |
| CRP (mg/l) | 0.83 (0.75, 0.92) | 0.001 |

Values in parentheses are 95 per cent confidence intervals. AUC, area under the receiver operating characteristic (ROC) curve; SIRS, Systemic Inflammatory Response Syndrome; BISAP, Bedside Index for Severity in Acute Pancreatitis (BISAP); APACHE, Acute Physiology And Chronic Health Evaluation; SOFA, Sequential Organ Failure Assessment; CRP, C-reactive protein; IL, interleukin.

**Table S2** Comparison of predictive values of the most effective predictors for major infection since admission

|  | Cut-off value | Sensitivity (%) | Specificity (%) | PPV (%) | NPV (%) | PLR | NLR | PP (%) |
| --- | --- | --- | --- | --- | --- | --- | --- | --- |
| Major infection (prevalence 3.8%) |  |  |  |  |  |  |  |  |
| BISAP (< 24 h) | ≥ 2 | 75.0 | 80.5 | 12.2 | 98.9 | 3.8 | 0.31 | 13.1 |
| Circulating histones (< 24 h) | ≥ 5.4 μg/ml | 44.4 | 88.1 | 12.9 | 97.6 | 3.7 | 0.63 | 12.8 |
| Urea (48 h) | ≥ 8 mmol/l | 66.7 | 91.4 | 24.0 | 98.5 | 7.8 | 0.37 | 23.6 |
| CRP (48 h) | ≥ 250 mg/l | 77.8 | 77.7 | 13.0 | 98.8 | 3.5 | 0.29 | 12.2 |

PPV, positive predictive value; NPV, negative predictive value; PLR, positive likelihood ratio; NLR, negative likelihood ratio; PP, post-test probability based on the test result being above the cut-off value. BISAP, Bedside Index for Severity in Acute Pancreatitis; CRP, C-reactive protein.
